# ROCK signaling at the crossroads of redox stress, mitochondrial dynamics, and metabolic disease

**DOI:** 10.1016/j.redox.2026.104109

**Published:** 2026-03-03

**Authors:** Yosuke Nagai, Keiichiro Matoba, Rimei Nishimura

**Affiliations:** Division of Diabetes, Metabolism, and Endocrinology, Department of Internal Medicine, The Jikei University School of Medicine, Tokyo, Japan

**Keywords:** ROCK1, Redox signaling, Mitochondrial dynamics, Metabolic remodeling, Cardiometabolic diseases

## Abstract

Rho-associated coiled-coil-containing kinases (ROCK1 and ROCK2) serve as central molecular switches that couple cytoskeletal dynamics with redox regulation and mitochondrial quality control. Dysregulated ROCK signaling promotes mitochondrial fragmentation, oxidative stress, and metabolic inflexibility, thereby linking nutrient overload to multi-organ dysfunction in diabetes, obesity, and cardiometabolic disease. Recent advances have identified ROCK1 as a key regulator of mitochondrial dynamics and bioenergetics: ROCK1 directly phosphorylates the fission protein Drp1 and suppresses the AMPK-PGC-1α pathway, resulting in impaired fatty acid oxidation, decreased mitochondrial biogenesis, and enhanced oxidative injury. Pharmacological or genetic inhibition of ROCK restores mitochondrial structure, energy metabolism, and redox balance across the heart, kidney, and liver, underscoring its therapeutic relevance. In contrast, ROCK2 plays more complementary roles in immune regulation and fibrotic remodeling, as evidenced by the clinical success of selective ROCK2 inhibition. In addition, metabolic drugs such as statins and GLP-1 receptor agonists can indirectly attenuate ROCK activity, suggesting feasible translational strategies for cardiometabolic disease. Despite these advances, isoform-specific mechanisms remain incompletely defined, and selective ROCK1 inhibitors have not yet been developed. Future studies should focus on clarifying ROCK1-specific signaling in mitochondrial homeostasis, developing tissue-targeted inhibitors, and combining ROCK modulation with metabolic or antioxidant therapies. A further understanding of the ROCK-mitochondria axis will enable the design of precise interventions to restore redox equilibrium and prevent progression of metabolic and cardiovascular disorders.

## Graphical abstract

**Figure:**
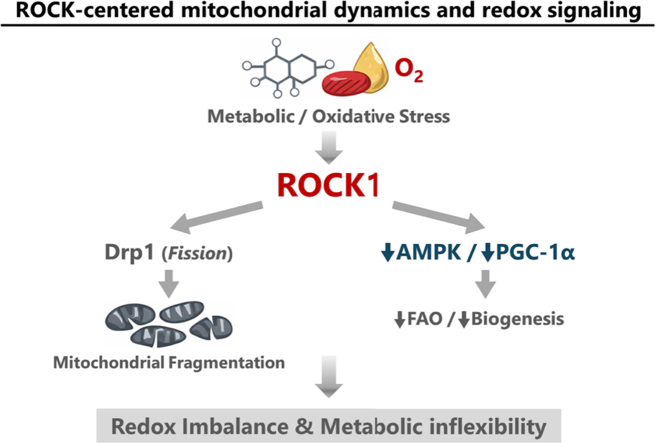

## 1 Introduction

Mitochondrial dysfunction together with redox imbalance represent core pathological signatures of metabolic diseases such as obesity, diabetes, fatty liver disease, and heart failure, linking nutrient overload to progressive organ injury [1]. When metabolic inputs exceed cellular capacity, excessive flux through β-oxidation and the tricarboxylic acid cycle burdens the electron transport chain, causing electron leakage at Complexes I and III and leading to overproduction of reactive oxygen species (ROS). Although physiological ROS serve crucial signaling roles, persistent excess outpaces antioxidant defenses, resulting in oxidative modification of mitochondrial lipids, proteins, and DNA, and ultimately diminishing ATP generation. At the same time, disruption of the NAD^+^/NADH ratio reduces the activity of sirtuins, AMPK, and PGC-1α, thereby attenuating mitochondrial biogenesis and antioxidant response systems [1,2].

Across different organs, diverse yet converging forms of mitochondrial injury have been observed, defining a unifying “redox-mitochondria axis.” In cardiomyocytes, suppression of fatty-acid oxidation together with AMPK inhibition provokes mitochondrial fragmentation and metabolic inflexibility, which in turn lead to contractile dysfunction [3]. In hepatocytes, surplus lipid flux and de novo lipogenesis amplify ROS-mediated lipid peroxidation and mitochondrial DNA injury, hastening the shift from simple steatosis to steatohepatitis [3]. In kidney proximal tubules—cells that rely heavily on oxidative phosphorylation—hyperglycemia and enhanced sodium reabsorption induce local hypoxia, Drp1-dependent mitochondrial fission, and reduced PGC-1α-driven biogenesis, preceding the onset of glomerular lesions [4]. Likewise, mitochondrial oxidative stress within vascular and adipose tissues interferes with nitric oxide signaling, endothelial function, and thermogenic adaptation, thereby connecting systemic insulin resistance with disturbed redox equilibrium [2].

Collectively, mitochondrial dysfunction and redox imbalance create a self-reinforcing feedback loop driving multi-organ metabolic decline. Enhanced ROS formation, defective oxidative metabolism, and NAD^+^ depletion operate as interconnected mechanisms that translate local cellular distress into systemic pathology. Notably, experimental data indicate that mitochondrial injury is at least partially reversible; therapeutic modulation of mitochondrial redox homeostasis—exemplified by inhibition of ROCK signaling—can help restore energetic and metabolic balance across organs [5,6].

Despite extensive characterization of mitochondrial redox imbalance in cardiometabolic disorders, the upstream signaling mechanisms that coordinate cytoskeletal remodeling, metabolic regulation, and oxidative stress responses remain incompletely defined. Defining such integrative pathways will therefore be essential for the development of precision redox-based interventions in cardiometabolic disease.

The RhoA-Rho-associated coiled-coil containing kinase (ROCK) pathway represents a pivotal signaling module that links mechanical forces and metabolic stress to the control of cellular architecture and function. RhoA, a small GTPase belonging to the Ras superfamily, functions as a molecular switch that cycles between GDP-bound inactive and GTP-bound active conformations, under the tight regulation of guanine nucleotide exchange factors (GEFs), GTPase-activating proteins (GAPs), and dissociation inhibitors (GDIs). Once activated, RhoA interacts with the Rho-binding domain of ROCK, which releases its autoinhibitory state and triggers phosphorylation of downstream substrates including myosin light chain (MLC), myosin phosphatase target subunit (MYPT1), LIM kinases, and ezrin/radixin/moesin proteins. These events collectively coordinate actomyosin contractility and cytoskeletal tension [7].

Beyond cytoskeletal dynamics, the RhoA-ROCK axis has gained recognition as a central mediator of oxidative and metabolic stress responses. A wide spectrum of stimuli—such as angiotensin II, endothelin-1, pro-inflammatory cytokines, and reactive oxygen species—can activate RhoA and ROCK across key cardiometabolic tissues, such as vascular endothelium, cardiomyocytes, hepatocytes, kidney parenchymal cells, and adipose tissue. In these settings, ROCK activation promotes oxidative stress through stimulation of NADPH oxidase and inhibition of endothelial nitric oxide synthase (eNOS), thereby exacerbating vasoconstriction, inflammation, and redox imbalance [8]. In metabolically active tissues, sustained ROCK signaling further suppresses AMPK and PGC-1α activity, leading to mitochondrial dysfunction, reduced fatty-acid oxidation, and impaired metabolic flexibility [9]. Given the established role of AMPK in promoting autophagy and mitochondrial quality control, ROCK-mediated suppression of AMPK may also contribute to impaired autophagic and mitophagic responses. This may facilitate the accumulation of damaged mitochondria and enhance mitochondrial ROS production under chronic metabolic stress.

Accumulating evidence points to RhoA-ROCK signaling as a central integrator of mitochondrial function and redox balance throughout cardiovascular and metabolic systems. In the ductus arteriosus (DA), oxygen-triggered constriction at birth depends not only on Ca^2+^ influx but also on mitochondrial ROS-driven activation of ROCK. This activation increases RhoB expression and maintains vasoconstriction through Ca^2+^ sensitization, even when extracellular calcium is lacking. Pharmacological inhibition of ROCK using Y-27632 or fasudil blocks this oxygen-induced constriction, indicating that mitochondrial redox signaling activates ROCK to sustain vascular tone during postnatal adaptation [10].

Conversely, excessive activation of the RhoA–ROCK cascade induces pathological mitochondrial fission and remodeling, thereby precipitating mitochondrial dysfunction and apoptosis in cardiomyocytes. RhoA overexpression enhances the pro-apoptotic molecule Bax via p53, stimulates caspase-9, and promotes cytochrome c-dependent cell death. Inhibition of ROCK abolishes these events, suggesting the participation of a mitochondrial death pathway [11]. Consistent with this, pharmacologic ROCK blockade by fasudil shields against lipopolysaccharide-induced myocardial dysfunction by reducing phosphorylation of Drp1 and cofilin, restoring F-actin structure, lowering oxidative stress, and promoting autophagic clearance of impaired mitochondria [12].

Comparable processes are evident in metabolic tissues. In diabetic nephropathy, ROCK activation induces Drp1-dependent mitochondrial fragmentation in podocytes, whereas ROCK deficiency maintains mitochondrial architecture and prevents albuminuria—thereby connecting hyperglycemia to organelle malfunction through ROCK signaling [13]. In vascular-injury settings, partial inhibition of ROCK dampens neointimal formation, vascular smooth-muscle proliferation, and leukocyte recruitment, underscoring its importance as a major driver of inflammation and tissue remodeling [14].

Taken together, these studies identify ROCK as a unifying upstream regulator that connects mitochondrial dynamics, redox equilibrium, and energetic control across cardiovascular and metabolic contexts. This conceptual framework provides the rationale for examining ROCK-driven redox and metabolic pathways in cardiometabolic disease.

## 2 Molecular mechanisms connecting ROCK to mitochondrial redox homeostasis

### 2.1 ROCK activation under oxidative and metabolic stress

Under physiological conditions, ROCK signaling coordinates cytoskeletal tension, vascular tone, and intracellular trafficking in response to dynamic mechanical and metabolic demands, thereby allowing cells to adapt mitochondrial positioning and energy production to fluctuating energetic needs. However, when persistently activated under chronic oxidative or metabolic stress, this adaptive signaling shifts toward sustained mitochondrial dysfunction and redox imbalance. Accordingly, across metabolic and cardiovascular diseases, RhoA–ROCK signaling has emerged as a common molecular denominator linking oxidative stress to mitochondrial and redox dysregulation through conserved downstream mechanisms that operate across tissues.

Oxidative and metabolic stress converge upon the RhoA-ROCK pathway in both experimental models and human disease. Among patients with type 2 diabetes and metabolic syndrome, ROCK activity—assessed by phosphorylation of MYPT1 or MBS in circulating leukocytes—is substantially elevated and shows a strong positive correlation with fasting glucose, triglyceride levels, and systemic inflammatory status [15,16]. This enhanced ROCK signaling often persists even after metabolic or antihypertensive interventions, and its elevation coincides with increased circulating malondialdehyde and angiotensin II, implying that oxidative stress together with renin-angiotensin activation act synergistically to augment ROCK activity.

Importantly, elevated leukocyte ROCK activity serves as an independent predictor of adverse cardiovascular outcomes—including stroke, revascularization, and cardiovascular mortality—highlighting its clinical relevance as a biomarker of systemic vascular risk [17]. Comparable activation patterns are observed in chronic kidney disease, cigarette smoking, and aging, where ROCK activity parallels indices of oxidative stress and inversely associates with endothelial function [[18], [19], [20]]. Notably, increased phosphorylated MYPT1 levels are also detected in kidney tissues from patients with diabetic kidney disease, indicating that ROCK activation accompanies local oxidative injury [21].

Collectively, these observations suggest that ROCK activation reflects cumulative oxidative burden across tissues rather than a disease- or organ-restricted phenomenon.

### 2.2 ROCK-mediated control of mitochondrial dynamics

Emerging evidence identifies mitochondrial fission as a central downstream event of pathological ROCK activation. A conserved mechanistic axis involves RhoA-dependent ROCK activation followed by phosphorylation of Drp1 at regulatory serine residues, including Ser600 and Ser616. ROCK-mediated Drp1 phosphorylation promotes its recruitment to the mitochondrial outer membrane, enhancing mitochondrial fragmentation and thereby increasing reactive oxygen species (ROS) production, impairing bioenergetics, and predisposing cells to apoptosis.

This ROCK-Drp1 signaling cascade has been documented across metabolic and inflammatory stress conditions. Under hyperglycemic stress, ROCK phosphorylates Drp1 at Ser600, driving excessive mitochondrial division and cellular injury in diabetic nephropathy models [13,22,23]. Similarly, ischemia-reperfusion injury activates transcriptional and receptor-mediated pathways—including KLF4-dependent ROCK upregulation and S1PR2 signaling—that amplify Drp1 phosphorylation and mitochondrial fragmentation, exacerbating oxidative stress and cell death [24,25]. Inflammatory triggers such as TLR4 activation or TNF-α signaling likewise stimulate ROCK-dependent phosphorylation of Drp1 at Ser616, leading to mitochondrial dysfunction in sepsis-associated organ injury [26,27]. Comparable mechanisms have been observed in neurodegenerative disease models, where ROCK activation enhances Drp1-dependent mitochondrial division and neuronal apoptosis [28].

Importantly, genetic or pharmacologic inhibition of ROCK consistently restores mitochondrial morphology, suppresses ROS accumulation, and improves cellular survival across these models. Collectively, these findings establish Drp1-dependent mitochondrial fission as a conserved downstream effector of ROCK signaling, integrating cytoskeletal tension and inflammatory signaling into a unified mitochondrial stress response rather than representing organ-specific pathology.

### 2.3 ROCK regulation of bioenergetics and antioxidant signaling

Beyond its classical role in cytoskeletal organization, ROCK functions as a key metabolic regulator that integrates mitochondrial energetics with redox control. A conserved mechanistic axis emerging across studies is the suppression of the AMPK–PGC-1α pathway by sustained ROCK activation. By inhibiting AMPK activity, ROCK attenuates mitochondrial biogenesis, reduces fatty-acid oxidation (FAO), and impairs adaptive substrate utilization, thereby promoting lipid accumulation and oxidative stress.

This ROCK–AMPK–PGC-1α circuit has been validated in multiple metabolic contexts. In hepatic models, ROCK activation suppresses AMPK and downregulates PGC-1α, fostering de novo lipogenesis and insulin resistance, whereas genetic deletion of ROCK enhances thermogenic capacity and energy expenditure [14]. Similarly, in diabetic kidney tissues, loss of ROCK preserves mitochondrial structure, restores PGC-1α and CPT1 expression, and improves FAO, while pharmacologic ROCK inhibition reactivates AMPK and normalizes mitochondrial function [29,30]. Comparable suppression of AMPK signaling has also been reported under chronic lipid overload or hyperglycemia in skeletal muscle [31].

In parallel with its interaction with AMPK, ROCK engages transcriptional amplifiers that reinforce metabolic and redox imbalance. FOXO1 directly promotes ROCK transcription in diabetic vascular endothelium and septic epithelial models, establishing a feed-forward loop that enhances Drp1 activation and oxidative stress [32,33]. Under hypoxic conditions, ROCK stabilizes HIF-1α by inhibiting prolyl hydroxylase–dependent degradation, thereby amplifying fibrotic and oxidative pathways [34].

Collectively, these findings position ROCK as a central integrator of bioenergetic rigidity and redox amplification. Through coordinated suppression of the AMPK–PGC-1α axis and activation of FOXO1/HIF-1α signaling programs, sustained ROCK activation disrupts mitochondrial homeostasis across metabolic and vascular systems rather than exerting organ-restricted effects.

### 2.4 Isoform-specific mechanism: ROCK1 vs. ROCK2

ROCK exists as two isoforms, ROCK1 (ROKb) and ROCK2 (ROKa), which share ≈ 65 % amino-acid identity yet differ markedly in structural organization, tissue distribution, and physiological function. Both isoforms contain an N-terminal kinase domain, a central coiled-coil region harboring the Rho-binding domain (RBD), and a C-terminal pleckstrin-homology (PH) domain interrupted by a cysteine-rich C1 region. However, their activation mechanisms diverge substantially: ROCK1 is cleaved and activated by caspase-3, whereas ROCK2 is cleaved by granzyme B—indicating context-dependent activation under apoptotic or cytotoxic stimuli [35]. Given that ROCK1 activation also promotes mitochondrial dysfunction, it is plausible that caspase-3-mediated ROCK1 activation forms a positive feedback loop amplifying apoptotic signaling.

Isoform-specific expression and subcellular localization further distinguish their functions. ROCK1 is enriched in non-neuronal tissues such as lung, liver, and kidney and primarily associates with the plasma membrane and actin cytoskeleton, governing cell contraction and mitochondrial dynamics. By contrast, ROCK2 is highly expressed in brain, heart, and vascular tissues and often localizes to the nucleus, where it interacts with transcriptional co-regulators such as p300 to modulate histone acetylation and gene expression [36]. Consistent with these patterns, ROCK1-deficient mice exhibit umbilical hernia and eyelid closure defects [37], whereas ROCK2-deficient mice display placental insufficiency, a prothrombotic phenotype, and intrauterine growth retardation [38]—highlighting their non-redundant roles in development.

Functionally, ROCK1 serves as a central modulator of metabolic and oxidative stress responses. It suppresses AMPK-PGC-1α signaling and fatty-acid oxidation, thereby inducing mitochondrial dysfunction and redox imbalance in models of diabetic nephropathy, fatty liver, and cardiac injury [13,14,27,29,39].

In contrast, ROCK2 primarily governs transcriptional, inflammatory, and immune remodeling programs. Through modulation of JNK/ERK, NF-κB, and STAT3/STAT5 signaling pathways, ROCK2 amplifies pro-inflammatory and fibrotic gene expression, contributing to chronic immune activation and tissue remodeling [[40], [41], [42], [43]]. Consistent with this role, selective ROCK2 inhibition attenuates pathogenic T follicular helper cell differentiation and improves autoimmune conditions such as chronic graft-versus-host disease and systemic lupus erythematosus.

Beyond immune regulation, ROCK2 also influences mitochondrial quality control and stress signaling. ROCK2 activation enhances p38 MAPK–dependent inflammatory responses and impairs mitophagy, whereas its inhibition restores mitochondrial homeostasis across models of neuroinflammation and vascular dysfunction [[44], [45], [46], [47]]. These observations collectively indicate that ROCK2 integrates transcriptional reprogramming with mitochondrial and inflammatory signaling rather than functioning in an organ-specific manner.

**Fig. 1:**
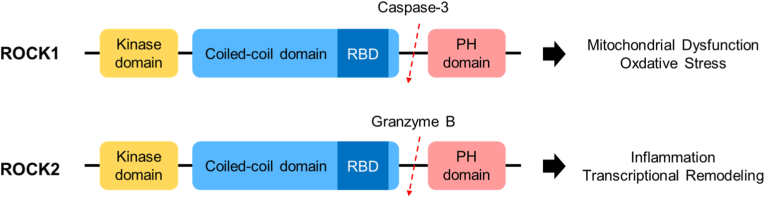
ROCK Isoforms ROCK1 and ROCK2. This schematic illustrates how the two Rho-associated kinases exert isoform-specific downstream functions. Under metabolic or oxidative stress, ROCK1 preferentially remodels mitochondrial structure and function, primarily through Drp1 activation and suppression of the AMPK–PGC-1α pathway, resulting in impaired fatty-acid oxidation and enhanced oxidative stress. In contrast, ROCK2 mainly controls transcriptional programs related to inflammation, fibrosis, and cytoskeletal organization, underscoring the complementary yet nonredundant roles of these isoforms in disease pathophysiology. PH; pleckstrin-homology, RBD; Rho-binding protein.

Taken together, ROCK1 primarily functions as a sensor of metabolic and oxidative stress, whereas ROCK2 predominantly governs transcriptional and immune remodeling programs (Fig. 1 and Table 1). Their structural and functional divergence supports complementary pathogenic roles across metabolic and cardiovascular diseases and underscores the importance of isoform-selective ROCK inhibition as a therapeutic strategy.

**Table 1:** **Mechanistic overview of ROCK-dependent pathways regulating mitochondrial redox homeostasis.** AMPK; AMP-activated protein kinase, DKD; diabetic kidney disease, Drp1; dynamin-related protein 1, FAO; fatty acid oxidation, FOXO1; forkhead box O1, I/R; ischemia-reperfusion, MASLD; metabolic dysfunction-associated steatotic liver disease, NF-κB; nuclear factor-κB, PGC-1α; peroxisome proliferator-activated receptor gamma coactivator-1 alpha, STAT; signal transducer and activator of transcription.

Mechanism	Upstream trigger	Downstream effector	Mitochondrial/Redox consequence	Representative context
**ROCK1-Drp1 axis**	Oxidative stress, cytokines, I/R	Drp1 phosphorylation	Mitochondrial fission, ↑ROS, apoptosis	DKD, I/R injury, sepsis, neurodegeneration
**ROCK1-AMPK/PGC-1α axis**	Metabolic overload, lipid excess	AMPK inhibition → ↓PGC-1α	↓FAO, impaired biogenesis, oxidative stress	MASLD, DKD, skeletal muscle insulin resistance
**FOXO1-ROCK1 feed-forward loop**	Hyperglycemia, inflammatory stress	↑ROCK transcription	Drp1 activation, endothelial barrier dysfunction	Diabetic endothelium, sepsis models
**ROCK2-NF-κB/STAT axis**	Immune activation	Transcriptional reprogramming	Fibrotic gene expression, inflammatory amplification	Autoimmunity, vascular inflammation

## 3 The-ROCK-mitochondria-redox axis in cardiometabolic disorders

### 3.1 Heart: mitochondrial dynamics and oxidative remodeling

In addition to cardiometabolic remodeling, ROCK-mediated mitochondrial dysregulation has been implicated in diverse forms of cardiac injury, including ischemia–reperfusion stress, inflammatory cardiomyopathy, chemotherapy-associated cardiotoxicity, and post-infarction remodeling.

ROCK functions as a central regulator of cytoskeletal tension, mitochondrial stability, and cardiac cell fate. In the ductus arteriosus (DA), oxygen-triggered constriction at birth depends not only on calcium influx but also on mitochondrial ROS-driven activation of ROCK, which maintains vasoconstriction through calcium sensitization even in the absence of extracellular calcium [10]. This mechanism first revealed a direct link between mitochondrial redox signaling and ROCK activation in cardiovascular adaptation.

Pathological activation of the RhoA-ROCK-Drp1 axis represents a fundamental driver of mitochondrial fragmentation and cardiomyocyte damage. During inflammatory stress—such as sepsis or TNF-α exposure—ROCK-dependent phosphorylation of Drp1 at Ser616 enhances mitochondrial fission, ROS overproduction, and cell death, whereas inhibition by fasudil or Y-27632 restores mitochondrial shape and cardiac performance [12,27]. Activation of RhoA by sphingosine-1-phosphate (S1P) similarly induces Drp1 phosphorylation and transient mitochondrial fragmentation, acting as a short-term adaptive response to oxidative stress [48].

At the transcriptional level, loss of KLF4 during ischemia/reperfusion promotes ROCK1 expression, augmenting Drp1-mediated mitochondrial fission, ROS formation, and apoptosis. Restoration of KLF4 normalizes mitochondrial architecture and cardiac performance, thereby identifying ROCK1 as a transcriptional effector of ischemic injury [24]. Conversely, Rnd3 binds directly to and inhibits ROCK1, preventing Drp1 phosphorylation at Ser616 and suppressing doxorubicin-induced mitochondrial fragmentation and PANoptosis. Cardiomyocyte-specific Rnd3 overexpression improves cardiac function and establishes ROCK1 as a downstream target of Rnd3-mediated cardioprotection [39]. Taken together, these findings define ROCK1 as a bidirectional regulator of mitochondrial dynamics—harmful when excessively activated, but counteracted by endogenous Rnd3.

ROCK signaling further integrates metabolic and structural remodeling within the myocardium. Under chronic hypoxia, the AMPK-FOXO1-ROCK1 cascade increases mitochondrial ROS generation and disrupts mitochondrial quality control, whereas miR-27b-3p-mediated FOXO1 repression confers protection [49]. Loss of lipid phosphate phosphatase-3 triggers Rho-ERK activation and impairs mitochondrial respiration [50]. Proteomic analyses of failing hearts have revealed enhanced ROCK1-vimentin signaling, linking cytoskeletal remodeling to mitophagy and mitochondrial renewal [51]. Likewise, RhoA-ROCK-dependent phosphorylation of N-Myc activates Parkin-mediated mitophagy, thereby preventing mitochondrial accumulation and senescence [52]. These findings may appear paradoxical in light of the pro-fragmentation and ROS-promoting effects described above. However, accumulating evidence suggests that ROCK1 exerts context- and stage-dependent effects on mitochondrial quality control. While sustained ROCK1 activation under chronic metabolic or ischemic stress promotes mitochondrial fragmentation and dysfunction, transient or compensatory activation may facilitate Parkin-dependent mitophagy to limit mitochondrial damage. Such differences likely reflect variations in temporal dynamics, disease severity, and cellular context rather than mutually exclusive roles of ROCK1. Downstream of Kif23, ROCK1 suppresses Ces1d-mediated fatty-acid oxidation, leading to lipid accumulation, mitochondrial dysfunction, and fibroblast transdifferentiation during post-infarction fibrosis [53].

**Fig. 2:**
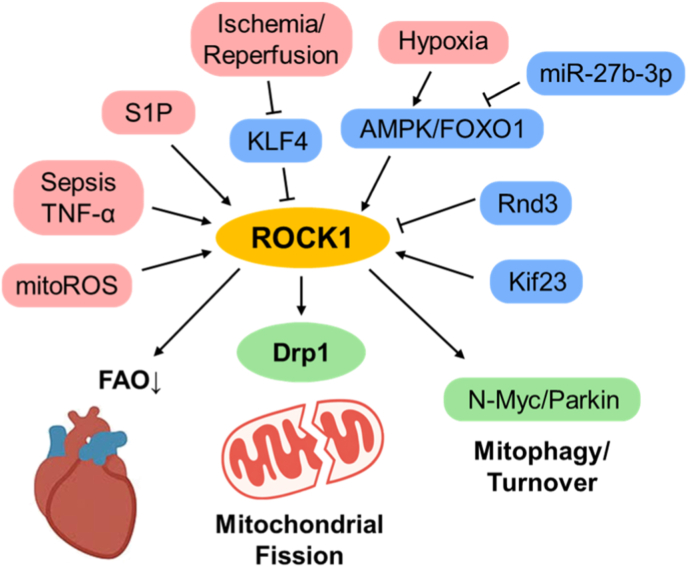
ROCK1-driven mitochondrial remodeling links cytoskeletal stress to cardiac redox injury. This schematic illustrates how sustained activation of ROCK1 in the heart promotes Drp1-dependent mitochondrial fragmentation, excessive mitochondrial ROS (mitoROS) production, and cardiomyocyte injury. Loss of KLF4 augments ROCK1 expression, whereas Rnd3 restrains ROCK1 activity, thereby preserving mitochondrial integrity. Beyond mitochondrial fission, ROCK1 signaling influences metabolic remodeling and mitophagy, integrating cytoskeletal stress with mitochondrial dysfunction in cardiac disease. AMPK; AMP-activated protein kinase, Drp1; dynamin-related protein 1, FOXO1; forkhead box O1, Kif23; kinesin family member 23, KLF4; krüppel-like factor 4, mitoROS; mitochondrial reactive oxygen species, N-Myc; neuroblastoma-derived Myc proto-oncogene protein, Rnd3; Rho family GTPase, TNF-α; tumor necrosis factor-alpha, S1P; sphingosine-1-phosphate.

Collectively, these studies identify ROCK1 as a pivotal coordinator of mitochondrial dynamics, bioenergetic regulation, and quality control in the heart under ischemic and failing conditions. The KLF4-ROCK1-Drp1 and Rnd3-ROCK1-Drp1 pathways constitute the most comprehensive mechanistic models of ROCK-driven mitochondrial injury and protection, integrating transcriptional, signaling, and organellar levels of cardiac pathophysiology (Fig. 2).

### 3.2 Vasculature: mitochondrial redox signaling and vascular remodeling

Within the vascular system, mitochondrial dysfunction serves as a central driver of ROCK-dependent oxidative injury. Early investigations demonstrated that mitochondrial ROS can activate RhoA/ROCK signaling to regulate vascular tone. In both human and murine vessels, acute glucose exposure transiently enhanced vascular constriction through mitochondrial-dependent yet RhoA-independent activation of ROCK in smooth muscle cells, leading to MYPT1 phosphorylation, inhibition of myosin light-chain phosphatase, and elevated contractile tone [54]. In rat tail arteries, cold-induced mitochondrial ROS promoted α_2_C-adrenoceptor translocation and vasoconstriction via RhoA/ROCK signaling [55]. Similarly, in mesenteric arteries, vasoconstrictors such as phenylephrine or high potassium triggered mitochondrial fission and ROS production, both prevented by ROCK inhibitors or the fission blocker mdivi-1 [56]. In hypertension, H_2_O_2_ and thromboxane A_2_ synergistically activated ROCK, establishing a redox-sensitive H_2_O_2_-TXA_2_-ROCK loop that amplified vascular hyperreactivity [57]. The uremic toxin indoxyl sulfate (IS) also induces mitochondrial- and NOX-derived ROS that activate ROCK and impair endothelial NO signaling, whereas fasudil or antioxidants restore vascular function [58]. Chronic hypoxia augments ROCK-dependent Ca^2+^ sensitization and HIF-1α-driven metabolic remodeling, contributing to pulmonary hypertension; dual inhibition of ROCK and HIF by emetine reverses these effects and improves right-ventricular function [59,60].

ROCK signaling additionally orchestrates vascular remodeling in response to hypertensive and injurious stimuli, largely through ROCK1-driven mitochondrial and redox mechanisms. Under angiotensin II stimulation, ROCK1 phosphorylates Drp1, causing excessive mitochondrial division, ROS generation, and phenotypic switching of vascular smooth muscle cells (VSMCs); these changes are prevented by the organosulfur compound diallyl trisulfide [61]. Downregulation of the inhibitory GTPase Rnd3 amplifies ROCK1-NOX signaling and mitochondrial superoxide formation, promoting hypertensive remodeling, whereas Rnd3 restoration reverses these changes and lowers blood pressure [62]. ROCK1 haploinsufficiency diminishes neointimal formation and inflammatory cell recruitment after vascular injury, underscoring its pathogenic role [63]. By contrast, ROCK2 primarily regulates contractile tone by binding to the myosin-binding subunit of myosin light-chain phosphatase (MLCP) [64] and cooperates with MK2 to promote the release of mitochondria-containing endothelial microparticles that propagate inflammation and hypertrophy [47]. Thus, ROCK1 mediates inflammatory and structural adaptation, while ROCK2 maintains cytoskeletal and contractile balance.

Endothelial mitochondrial injury represents another major outcome of ROCK activation. During sepsis, extracellular NAMPT activates RhoA-ROCK-Drp1 signaling, inducing mitochondrial fission, ROS accumulation, and barrier disruption; these events are blocked by inhibition of ROCK or Drp1 [26]. Under hyperglycemia, FOXO1 upregulation activates ROCK1-Drp1 signaling, resulting in mitochondrial fragmentation and endothelial apoptosis [32]. In diabetic wounds, ROCK1 interacts with RIPK4 to suppress AMPK phosphorylation, leading to elevated mitochondrial ROS and impaired angiogenesis, whereas fasudil restores AMPK-eNOS coupling and accelerates vascular repair [65]. Rnd3-Trim40-dependent degradation of ROCK1 further limits endothelial leakage and microvascular damage in diabetes [66], while GLP-1-based therapies improve endothelial function by suppressing the AGE/RAGE-ROCK–NF–κB pathway and activating AMPK [67].

ROCK signaling also contributes to oxidative endothelial injury in atherosclerosis and infection. Activation of ROCK drives mitochondrial-to-cytosolic translocation of arginase 2 through the LOX-1/MPP pathway, reducing nitric oxide bioavailability and promoting superoxide generation [68]. *Porphyromonas gingivalis* infection activates the RhoA-ROCK1-Drp1 axis, leading to mitochondrial DNA depletion and ATP loss, both of which are prevented by ROCK inhibition [69]. In obesity and diabetes, ROCK1-TGM2 signaling promotes hypertrophic remodeling of small arteries [70]. The TRPM8 channel maintains mitochondrial Ca^2+^ homeostasis and suppresses RhoA/ROCK activation during cold or angiotensin II stress [71]. Mitochondrial ROS also regulate actomyosin dynamics through ROCK to drive epithelial remodeling [72], and endothelial S1PR2 triggers ischemia/reperfusion-induced mitochondrial fragmentation and inflammasome activation via the Rho-ROCK1-Drp1 pathway [25].

**Fig. 3:**
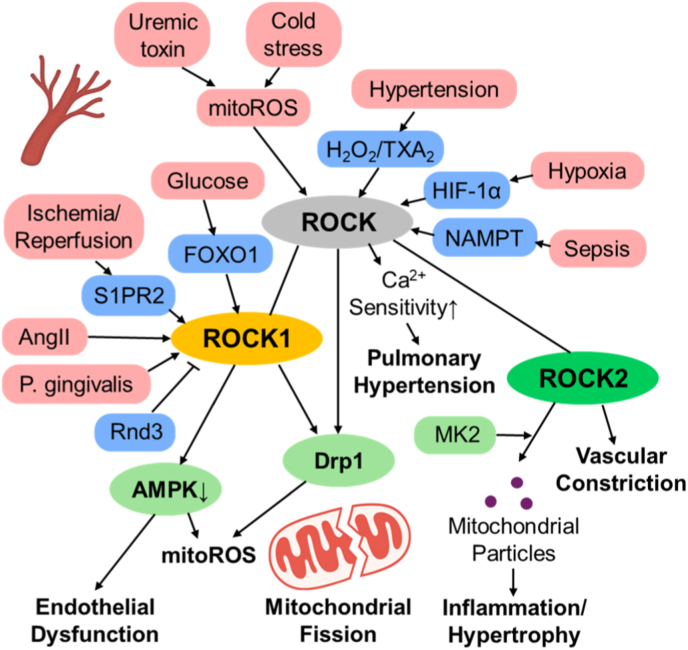
Isoform-specific ROCK signaling integrates mitochondrial ROS with vascular dysfunction. This schematic illustrates how mitochondrial reactive oxygen species (ROS) activate RhoA/ROCK signaling in the vasculature, thereby promoting increased vascular tone, inflammation, and structural remodeling. In endothelial cells, ROCK1 preferentially induces Drp1-dependent mitochondrial fission, leading to endothelial injury and redox imbalance. In contrast, ROCK2 primarily regulates cytoskeletal tension and contractile responses in vascular smooth muscle cells. Importantly, pharmacological or genetic ROCK inhibition restores mitochondrial integrity and improves vascular function, highlighting ROCK signaling as a central mediator linking mitochondrial redox stress to vascular disease. AMPK; AMP-activated protein kinase, AngII; angiotensin II, Drp1; dynamin-related protein 1, FOXO1; forkhead box O1, H_2_O_2_;hydrogen peroxide, HIF-1α; hypoxia-inducible factor-1 alpha, MK2; MAPK-activated protein kinase 2, NAMPT; nicotinamide phosphoribosyltransferase, Rnd3; Rho family GTPase 3, S1PR2; sphingosine-1-phosphate receptor 2.

In summary, these studies define a unifying model in which mitochondrial ROS activate RhoA/ROCK signaling to amplify vascular tone, inflammation, and remodeling. ROCK1 predominantly mediates inflammatory and structural responses, while ROCK2 governs cytoskeletal tension and contractile control. Together, they integrate mitochondrial stress with vascular homeostasis, and ROCK inhibition restores mitochondrial integrity, endothelial function, and tissue perfusion (Fig. 3).

### 3.3 Kidney: diabetic oxidative stress and defective FAO

In diabetic nephropathy, hyperglycemia promotes mitochondrial fragmentation in podocytes through ROCK1-dependent phosphorylation of Drp1 at Ser600, thereby driving mitochondrial fission and podocyte injury. Deletion of ROCK1 prevents Drp1 activation, preserves mitochondrial architecture, and protects against albuminuria, establishing ROCK1 as an upstream regulator that connects hyperglycemia with mitochondrial dysfunction and glomerular pathology [13]. Activation of the thromboxane/prostaglandin (TP) receptor further amplifies this process by enhancing ROCK1 expression and Drp1 phosphorylation, whereas inhibition of either the TP receptor or ROCK1 prevents mitochondrial fragmentation and podocyte loss—defining the TP-ROCK1-Drp1 axis as a receptor-mediated pathway of mitochondrial injury [22]. In experimental models of diabetic nephropathy, podocyte-specific deletion of the phosphatase SHP-1 decreases the expression of RhoA and ROCK1, restores slit diaphragm proteins, and prevents both albuminuria and fibrosis. These findings indicate that SHP-1 positively regulates the RhoA–ROCK1 axis in diabetic podocytes, thereby contributing to cytoskeletal disruption and mitochondrial injury [73]. Conversely, ROCK2 exerts distinct transcriptional effects: podocyte-specific ROCK2 ablation enhances PPARα-driven fatty-acid oxidation (FAO) and protects against glomerular damage, indicating that ROCK2 represses PPARα signaling under metabolic stress [74].

In mesangial cells, ROCK1 deficiency maintains mitochondrial integrity and FAO by reactivating the AMPK/PGC-1α axis and CPT1 expression, thereby reducing oxidative stress and glomerular injury [29]. In glomerular endothelial cells, hyperglycemia triggers the S1PR2-RhoA-ROCK1-Drp1 cascade, resulting in mitochondrial fragmentation, ROS overproduction, and apoptosis, while inhibition of S1PR2 or ROCK reverses these effects [75]. In tubular epithelial cells, stimulation of S1P_2_ similarly activates ROCK, inducing epithelial-mesenchymal transition (EMT) and contributing to fibrotic remodeling [76]. In these cells, Numb deficiency enhances ROCK-dependent Drp1 phosphorylation and mitochondrial fragmentation during ischemia-reperfusion or cisplatin-induced injury, whereas ROCK inhibition preserves mitochondrial morphology and function [77]. Similarly, high glucose activates RhoA/ROCK1/Drp1-dependent fission in proximal tubular cells, which is reversed by β_2_-adrenergic receptor stimulation that restores Mfn1 activity and mitochondrial respiration [23]. Noncoding RNA networks such as circPWWP2A-miR-182 signaling derepress ROCK1, increasing mitochondrial ROS and fibrosis, both reversed by ROCK1 inhibition [78]. Resveratrol displays dose-dependent actions—low doses activate SIRT1 and suppress ROCK1-mediated fibrosis, whereas higher doses augment mitochondrial ROS and EMT via ROCK1 signaling [79]. Furthermore, ROCK2 regulates mineralocorticoid receptor expression through the STAT3 pathway in tubules, linking hormonal signaling to metabolic and electrolyte balance in the kidney [80].

**Fig. 4:**
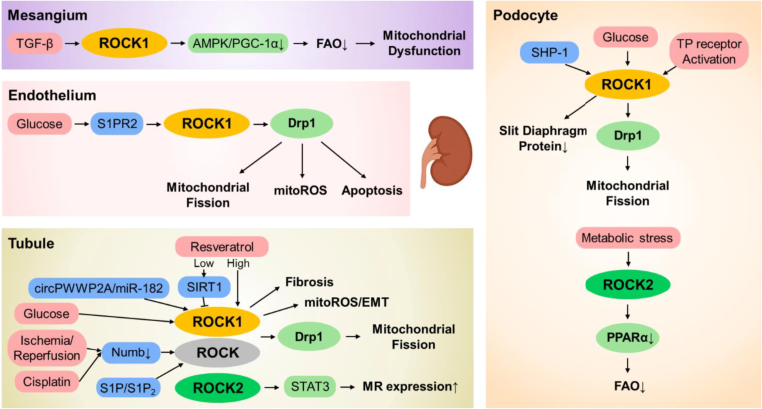
Isoform- and cell type–specific ROCK signaling coordinates mitochondrial dysfunction across kidney compartments. This schematic summarizes how ROCK isoforms exert distinct mitochondrial and metabolic effects across kidney cell types. In podocytes, ROCK1 promotes Drp1-mediated mitochondrial fission and cellular injury, whereas ROCK2 suppresses PPARα-dependent fatty-acid oxidation (FAO). In mesangial cells, ROCK1 inhibition preserves the AMPK–PGC-1α–CPT1 axis, thereby limiting oxidative stress. In glomerular endothelial cells, S1PR2–ROCK1–Drp1 signaling induces mitochondrial fragmentation and apoptosis. In tubular epithelial cells, ROCK1 activation enhances Drp1 phosphorylation, ROS generation, and fibrotic signaling, while pharmacological ROCK inhibition restores mitochondrial integrity. Collectively, this figure highlights ROCK1 as a dominant driver of mitochondrial injury across kidney compartments, with ROCK2 exerting complementary metabolic regulatory functions. AMPK; AMP-activated protein kinase, circPWWP2A; circular RNA PWWP domain-containing protein 2A, Drp1; dynamin-related protein 1, FAO; fatty acid oxidation, PGC-1α; peroxisome proliferator-activated receptor gamma coactivator-1 alpha, PPARα; peroxisome proliferator-activated receptor alpha, S1P; sphingosine-1-phosphate, S1P2; sphingosine-1-phosphate receptor 2, S1PR2; sphingosine-1-phosphate receptor 2, SHP-1; src homology region 2 domain-containing phosphatase-1, SIRT1; sirtuin 1, STAT3; signal transducer and activator of transcription 3, TGF-β; transforming growth factor-beta, TP; thromboxane/prostaglandin.

Overall, ROCK1 predominantly drives Drp1-dependent mitochondrial fission and oxidative stress across podocytes, mesangial cells, endothelial cells, and tubular compartments, whereas ROCK2 modulates transcriptional and hormonal metabolic responses through PPARα and STAT3. These complementary yet distinct roles converge to integrate mitochondrial dysfunction, fibrotic remodeling, and systemic metabolic dysregulation in diabetic nephropathy (Fig. 4).

### 3.4 Liver: steatosis, oxidative stress, and redox-sensitive lipogenesis

ROCK signaling plays a pivotal role in hepatic metabolic remodeling, integrating lipid accumulation, mitochondrial stress, and insulin resistance. In the liver, ROCK1 functions as a metabolic switch that promotes steatosis while suppressing energy expenditure. Liver-specific deletion of ROCK1 confers resistance to high-fat diet-induced steatosis through AMPK activation, whereas ROCK1 activation suppresses AMPK and enhances de novo lipogenesis. Mechanistically, cannabinoid receptor signaling has been shown to activate hepatic ROCK1, and a ROCK1/AMPK axis is required for cannabinoid-induced lipogenesis, thereby establishing a mechanistic link between endocannabinoid signaling, AMPK suppression, and fatty liver development [14]. ROCK1 also mediates hepatocellular stress communication: palmitate-induced activation of death receptor 5 (DR5) triggers ROCK1-dependent release of proinflammatory extracellular vesicles, propagating macrophage activation and inflammation in metabolic dysfunction-associated steatohepatitis (MASH); inhibition by fasudil markedly reduces hepatic injury and fibrosis [81].

Multiple noncoding RNA networks converge on ROCK1 to regulate hepatic lipogenesis. The circ_0057558-miR-206-ROCK1 circuit suppresses AMPK and promotes lipid deposition, whereas miR-206 mimics restore AMPK signaling and improve steatosis [82]. Similarly, the HOTAIR-miR-130b-3p-ROCK1 and NEAT1-miR-146a-5p-ROCK1 pathways drive lipid accumulation by releasing ROCK1 from miRNA inhibition, leading to AMPK inactivation and increased SREBP-mediated lipogenesis [83,84]. Collectively, these noncoding RNA-ROCK1 axes define a shared molecular framework linking post-transcriptional control to mitochondrial lipid stress.

ROCK2 exerts distinct functions in hepatic lipid and redox regulation. In lipid-loaded hepatocytes, ROCK2 colocalizes with lipid droplet-associated proteins and redox mediators such as HILPDA, HIF1α, and PDK4, suggesting its involvement in adaptive metabolic remodeling and mitochondrial stress signaling [85]. Recent findings indicate that lncRNA-driven ROCK2 activation suppresses mitophagy: the ZNF143-NEAT1-SND1-ROCK2 pathway stabilizes ROCK2 mRNA and inhibits mitochondrial clearance, whereas silencing NEAT1 or ROCK2 restores mitophagy and alleviates steatosis [86]. Likewise, the NORAD-miR-511-3p-ROCK2 axis directly stabilizes ROCK2 protein and amplifies inflammation, fibrosis, and β-cell dysfunction, underscoring ROCK2 as a key effector in systemic metabolic remodeling [87].

**Fig. 5:**
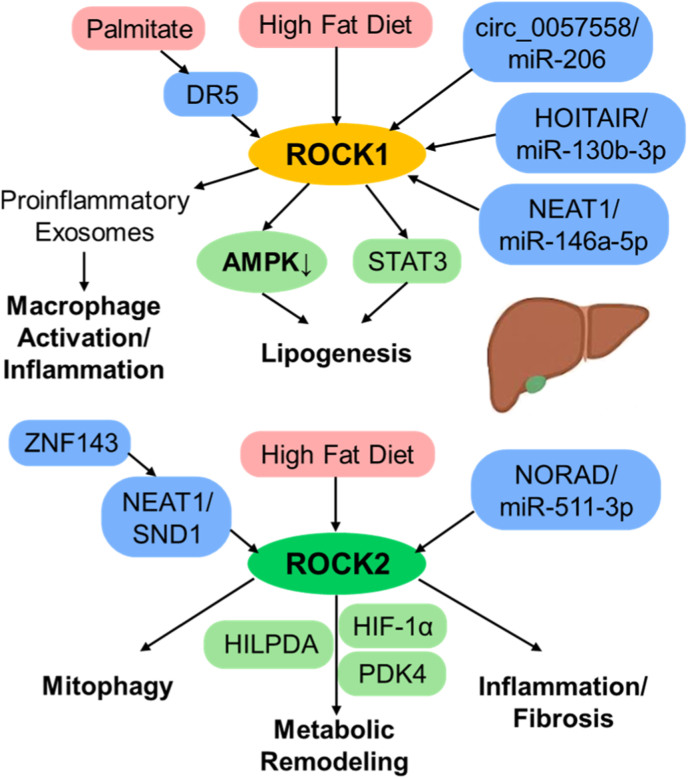
Differential ROCK signaling drives metabolic and redox remodeling in the liver. This schematic illustrates how ROCK-dependent signaling reprograms hepatic metabolism to promote steatosis, inflammation, and insulin resistance. ROCK1 suppresses AMPK activity, thereby enhancing de novo lipogenesis and the release of inflammatory extracellular vesicles (EVs). These effects are further amplified by non-coding RNA (ncRNA) networks, which reinforce ROCK1 signaling and accelerate lipid accumulation in hepatocytes. In parallel, ROCK2 regulates redox-sensitive lipid remodeling and inhibits mitophagy through lncRNA-mediated stabilization mechanisms, contributing to the persistence of dysfunctional mitochondria. Through these complementary pathways, both ROCK isoforms converge to impair insulin signaling and increase hepatic glucose output, establishing ROCK signaling as a central driver of metabolic dysfunction in the liver. AMPK; AMP-activated protein kinase, DR5; death receptor 5, HIF-1α; hypoxia-inducible factor-1 alpha, HILPDA; hypoxia-inducible lipid droplet-associated protein, HOTAIR; HOX transcript antisense intergenic RNA, NEAT1; nuclear paraspeckle assembly transcript 1, NORAD; non-coding RNA activated by DNA damage, PDK4; pyruvate dehydrogenase kinase 4, SND1; staphylococcal nuclease and tudor domain-containing 1, STAT3; signal transducer and activator of transcription 3, ZNF143; zinc finger protein 143.

Beyond lipid metabolism, hyperactivation of hepatic ROCK1/2 contributes to insulin resistance. Phosphoproteomic profiling of iPSC-derived hepatocytes from patients with type 2 diabetes revealed elevated ROCK1/2 activity as a driver of defective insulin signaling, whereas ROCK inhibition partially restored AKT phosphorylation and metabolic responsiveness [88]. Moreover, activation of the G12/13-ROCK1-JNK cascade enhances hepatic glucose production and hyperglycemia independently of insulin signaling, identifying ROCK1 as a redox-sensitive regulator of hepatic gluconeogenesis [89] (Fig. 5).

### 3.5 Adipose tissue: ROCK signaling in insulin resistance and adipose remodeling

ROCK signaling coordinates adipocyte lineage commitment, differentiation, and metabolic responsiveness through the coupling of cytoskeletal dynamics and transcriptional programs. During early adipogenesis, Rho-ROCK activation represses PPARγ and C/EBPα, diverting mesenchymal precursors toward myogenic differentiation via an IGF-1-dependent mechanism [90]. Remodeling of the actin cytoskeleton plays a crucial role in this process: disruption of stress fibers results in G-actin accumulation, which sequesters the transcriptional coactivator MKL1 within the cytoplasm, thereby allowing PPARγ expression and adipocyte differentiation [91]. Thus, dynamic modulation of Rho-ROCK signaling links actin architecture to transcriptional control during the formation of adipocytes. Isoform analyses further revealed that ROCK2, rather than ROCK1, acts as a principal brake on adipocyte differentiation and insulin signaling. ROCK2 deficiency enhances Akt phosphorylation, PPARγ induction, and insulin sensitivity, whereas high-fat diet feeding elevates ROCK2 activity and promotes inflammatory adipose remodeling [92,93]. These findings identify ROCK2 as a key suppressor of adipogenic and insulin-sensitizing transcriptional pathways.

By contrast, ROCK1 mainly regulates insulin receptor signaling and mitochondrial metabolism. Deletion of ROCK1 in adipocytes increases phosphorylation of the insulin receptor and IRS-1/PI3K/Akt pathway, improving insulin sensitivity without altering overall adiposity [94]. Mechanistically, ROCK directly phosphorylates IRS-1 at Ser632/635, thereby enhancing insulin-stimulated PI3K activation and defining a positive regulatory role in insulin signaling [95]. Systemically, high-fat diet-induced ROCK1 activation drives insulin resistance and macrophage infiltration, both of which are reversed by pharmacologic inhibition or by expression of dominant-negative RhoA [96]. In addition, NO/cGMP-dependent activation of protein kinase G (PKGI) facilitates brown adipocyte differentiation and mitochondrial biogenesis by suppressing RhoA-ROCK signaling, thereby restoring IRS-1-PI3K-Akt signaling and thermogenic UCP1 expression [97]. Circulating miR-324-5p further modulates this network by directly targeting ROCK1, suppressing AKT/GSK signaling and promoting lipid deposition [98]. In perivascular adipose tissue of obese *db/db* mice, activation of the mineralocorticoid receptor induces mitochondrial dysfunction and senescence via a SIRT-ROCK-p53 axis, impairing anticontractile function, while MR antagonists or mitochondrial antioxidants restore respiration and vascular tone [99]. Clinically, ROCK activity in leukocytes from individuals with metabolic syndrome and type 2 diabetes is elevated and correlates with adiposity, hyperglycemia, and systemic inflammation [15,21].

**Fig. 6:**
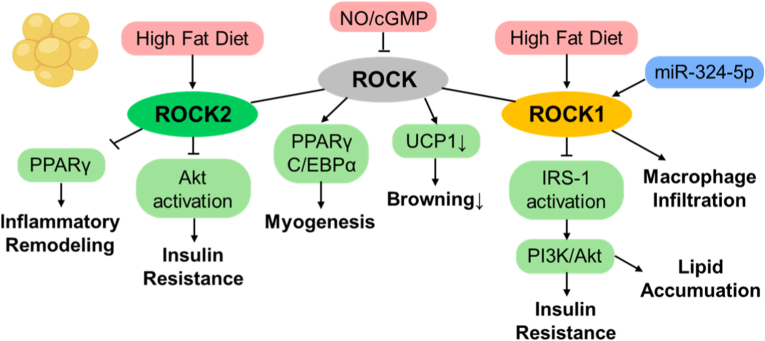
Coordinated ROCK signaling underlies inflammatory and metabolic dysfunction in adipose tissue. This schematic illustrates how ROCK signaling functions as a coordinated network in adipose tissue remodeling, with partial functional specialization between ROCK isoforms. While ROCK2 has been implicated in the inhibition of adipocyte differentiation and the promotion of inflammatory remodeling, and ROCK1 has been associated with impaired insulin signaling and altered mitochondrial metabolism, these pathways ultimately converge to disrupt adipose tissue function. Through such integrated effects, ROCK signaling links transcriptional programs and metabolic pathways, thereby contributing to adipose dysfunction and its endocrine impact on whole-body metabolism. Akt; protein kinase B, cGMP; cyclic guanosine monophosphate, C/EBPα; CCAAT/enhancer-binding protein alpha, IRS-1; insulin receptor substrate-1, NO; nitric oxide, PI3K; phosphoinositide 3-kinase, PPARγ; peroxisome proliferator-activated receptor gamma, UCP1; uncoupling protein 1.

Together, ROCK1 promotes insulin resistance and mitochondrial stress, whereas ROCK2 limits adipogenesis and facilitates inflammatory remodeling. These coordinated yet distinct actions position ROCK signaling as a central node that connects adipose dysfunction with systemic metabolic disease (Fig. 6).

### 3.6 Skeletal muscle: insulin signaling and mitochondrial adaptation

ROCK signaling regulates mitochondrial dynamics, energy metabolism, and insulin responsiveness in skeletal muscle through distinct yet complementary functions of ROCK1 and ROCK2. Under physiological conditions, ROCK1 serves as a positive modulator of insulin signaling. In ROCK1-deficient mice, insulin-stimulated PI3K-Akt activation and IRS-1 phosphorylation are reduced despite normal adiposity, indicating that basal ROCK1 activity is required for proper insulin responsiveness [100]. In pathological contexts, however, ROCK1 activity becomes detrimental: in subtotal-nephrectomy models, ROCK1 activation induces Drp1-dependent mitochondrial fission, oxidative stress, and muscle atrophy, whereas ROCK1 inhibition restores mitochondrial structure and respiration [101]. Constitutive activation of ROCK1 additionally suppresses the myokine irisin, thereby impairing muscle-adipose communication, thermogenic capacity, and systemic insulin sensitivity—all of which are rescued by exogenous irisin administration [102]. Clinically, insulin normally activates ROCK1 in human skeletal muscle, but this response is blunted in obesity and type 2 diabetes, where reductions in IRS-1 phosphorylation and glucose uptake occur; upregulation of RhoE contributes to this defect [103].

In contrast, ROCK2 mediates adaptive and compensatory responses within skeletal muscle. Physical exercise reactivates RhoA-ROCK2 signaling, enhances IRS-1 and Akt phosphorylation, and improves insulin sensitivity in obese mice [104]. Conversely, lipid overload activates the GGPPS-RhoA-ROCK-IRS1 axis, promoting serine phosphorylation of IRS-1 and contributing to insulin resistance [105].

**Fig. 7:**
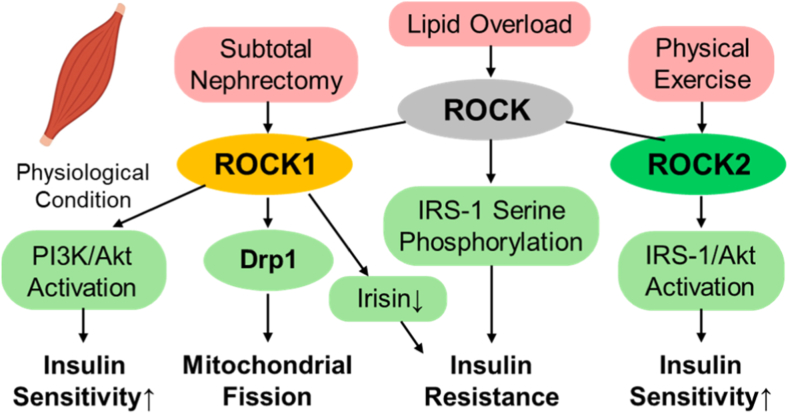
ROCK1- and ROCK2-dependent regulation of insulin signaling and mitochondrial adaptation in skeletal muscle. This schematic illustrates how ROCK1 and ROCK2 exert distinct and context-dependent roles in skeletal muscle insulin signaling and mitochondrial remodeling. Under basal conditions, ROCK1 supports physiological insulin signaling, whereas excessive ROCK1 activation promotes Drp1-dependent mitochondrial fission, oxidative stress, and reduced irisin secretion, thereby impairing muscle insulin sensitivity. In contrast, ROCK2 mediates adaptive metabolic responses, including exercise-induced enhancement of IRS-1 and Akt signaling, contributing to improved insulin responsiveness. Through these complementary actions, ROCK isoforms integrate cytoskeletal dynamics with mitochondrial and metabolic pathways that determine skeletal muscle insulin sensitivity. Akt; protein kinase B, Drp1; dynamin-related protein 1, IRS-1; insulin receptor substrate-1, PI3K; phosphoinositide 3-kinase.

Taken together, ROCK1 is indispensable for maintaining basal insulin signaling but becomes pathogenic when excessively activated, whereas ROCK2 functions as a restorative mediator that supports insulin sensitization and metabolic equilibrium in skeletal muscle (Fig. 7).

## 4 ROCK signaling in neural, sensory, and structural systems

### 4.1 Nervous system: energy balance and mitochondrial resilience

ROCK signaling integrates neuronal energy metabolism, mitochondrial dynamics, and neuroendocrine regulation. In the hypothalamus, ROCK1 serves as a crucial mediator of leptin-JAK2 signaling, linking energy sensing to body weight control. Neuron-specific deletion of ROCK1 in either POMC or AgRP neurons induces hyperphagia, obesity, and leptin resistance, confirming its essential role in leptin sensitivity and STAT3 activation [106,107]. The ROCK1-JAK2 interaction promotes phosphorylation of STAT3 and FOXO1, coordinating the anorexigenic and thermogenic effects of leptin. Conversely, diet-induced obesity suppresses hypothalamic ROCK1 activity, reducing energy expenditure and causing central leptin resistance, thereby identifying ROCK1 as a key molecular switch governing hypothalamic energy balance.

ROCK2, in contrast, mainly contributes to neuronal injury and mitochondrial dysfunction in neurodegenerative conditions. In Alzheimer's disease models, pharmacologic or natural inhibition of ROCK2 restores mitochondrial integrity and synaptic function. Qiangji Decoction downregulates ROCK2 and Drp1 phosphorylation while enhancing Mfn1/2 and OPA1, improving ATP production and cognitive performance [108]. Likewise, fasudil administration in the PS19 tauopathy model activates mitochondrial TCA cycle enzymes, enhances blood-brain barrier metabolism, and reduces tau phosphorylation, emphasizing the therapeutic promise of ROCK inhibition in tau-related neurodegeneration [109]. Exercise-induced activation of SIRT1 suppresses ROCK1 and increases ADAM10 and PGC-1α expression, thereby shifting APP processing toward the non-amyloidogenic pathway [110]. In Huntington's disease, inhibition of ROCK with fasudil or simvastatin preserves mitochondrial function and activates prosurvival signaling via the Akt/eNOS/PGC-1α pathway [111]. In temporal lobe epilepsy, ROCK activation promotes ASIC1a trafficking to mitochondria, resulting in oxidative damage and apoptosis, whereas ROCK inhibition or ASIC1a blockade restores neuronal viability [112]. Similarly, ROCK inhibitors enhance Parkin-mediated mitophagy through HK2 recruitment to mitochondria, promoting dopaminergic neuron survival in Parkinson's models [113]. Parallel studies show that RhoA/ROCK activation disrupts axonal mitochondrial transport via Ca^2+^-HDAC6-Miro1 signaling, while HDAC6 inhibition restores motility and axon elongation [114]. Altogether, ROCK inhibition emerges as a unifying therapeutic approach that restores mitochondrial resilience across diverse neurodegenerative settings.

In ocular tissues, ROCK inhibition reprograms mitochondrial metabolism and promotes regenerative potential. In corneal endothelial cells, ripasudil or Y-27632 enhances oxidative phosphorylation and AMPK activity, coupling mitochondrial respiration to cytoskeletal remodeling [115,116]. In retinal pigment epithelial cells, Y-27632 promotes tunneling nanotube-mediated mitochondrial transfer, restoring mitochondrial function following light-induced injury [117].

In summary, ROCK1 maintains hypothalamic energy regulation, whereas ROCK2 drives neuronal mitochondrial injury, with ROCK inhibition conferring neuroprotective and regenerative benefits across multiple neural contexts.

### 4.2 Other organs and systemic regulation

ROCK1 and ROCK2 distinctly regulate mitochondrial homeostasis and metabolic adaptation across multiple organ systems. ROCK1 primarily governs oxidative stress, mitochondrial activity, and energy coupling. In aging- and cellular senescence-related processes, inhibition of Rho-associated kinase by Y-27632 or fasudil rejuvenates mitochondrial respiration and chromatin organization, restoring proliferative capacity and accelerating wound repair [118]. In fibroblasts from Hutchinson-Gilford progeria syndrome, ROCK1 phosphorylates Rac1b, promoting its interaction with cytochrome *c* and driving mitochondrial ROS generation; ROCK blockade disrupts this complex, restores respiration, and alleviates nuclear abnormalities [119]. Likewise, in epidermal stem cells exposed to UV-B, Annexin A1 activates RhoA/ROCK1 signaling to sustain mitochondrial potential and mitophagy, whereas ROCK1 inhibition abolishes this protective effect [120].

In diabetic wound granulation tissue, particularly within vascular endothelial cells, overactive ROCK1 associates with RIPK4 to inhibit AMPK phosphorylation, diminishing eNOS activity and impairing angiogenesis, while fasudil reinstates AMPK-eNOS signaling and mitigates mitochondrial oxidative stress [65]. Loss of mTORC1 in epidermal keratinocytes or macrophages similarly provokes compensatory ROCK1 activation, leading to epithelial adhesion defects or defective efferocytosis; ROCK inhibition rescues both, linking metabolic cues to cytoskeletal and mitochondrial integrity [121,122]. In pancreatic β-cells, ROCK1 couples glycolysis with mitochondrial ATP generation through pyruvate-kinase interaction, ensuring efficient glucose-stimulated insulin release [123]. Within the respiratory system, obesity-driven GPR40-RhoA-ROCK1 activation fosters airway remodeling and inflammation in airway smooth-muscle cells, reversible through GPR40 blockade [124]. The HDAC3-FOXO1-ROCK1 axis further disrupts mitochondrial fusion and fatty-acid oxidation in alveolar epithelial cells, whereas HDAC3 inhibition restores mitochondrial balance [32]. By contrast, ROCK2 contributes to structural remodeling and mitochondrial quality control within connective and skeletal cell populations, including osteoclasts and chondrocytes. In osteoclasts, deletion of Gα13 leads to hyperactivation of RhoA-ROCK2 signaling, enhancing cytoskeletal reorganization and mitochondrial biogenesis that drive bone resorption and inflammatory osteolysis [125]. In osteoarthritic cartilage, impaired FUNDC1-mediated mitophagy causes mitochondrial dysfunction and chondrocyte loss; selective ROCK2 inhibition reactivates FUNDC1, improves mitochondrial quality, and prevents cartilage degradation [126].

Collectively, these findings indicate that ROCK1 predominates in oxidative and metabolic stress responses, whereas ROCK2 preserves structural integrity and mitophagic balance—cooperatively maintaining systemic energy and tissue homeostasis.

## 5 Therapeutic modulation of ROCK-mitochondrial-redox axis

### 5.1 Pharmacological ROCK inhibitors in metabolic and vascular disease

Currently available Rho-associated kinase (ROCK) inhibitors include the dual ROCK1/2 blockers Y-27632, fasudil, ripasudil, and netarsudil, along with the selective ROCK2 inhibitor belumosudil [127]. Among these, fasudil is approved in Japan and China for preventing delayed cerebral ischemia and vasospasm after subarachnoid hemorrhage [128], whereas ripasudil and netarsudil are marketed as ophthalmic formulations for glaucoma and corneal endothelial disorders [129].

Clinical evidence has established that the RhoA/ROCK pathway plays a pivotal role in human cardiovascular disease. In patients with pulmonary arterial hypertension, ROCK activity is markedly increased, and both intravenous and oral administration of fasudil significantly reduces pulmonary vascular resistance [130,131]. Similarly, intracoronary fasudil effectively suppresses coronary artery spasm in vasospastic angina, directly implicating ROCK activation in coronary hyperconstriction [132]. Elevated ROCK expression in human atherosclerotic and inflammatory vascular lesions further underscores its pathogenic contribution to vascular remodeling [133].

Beyond cardiovascular disease, fasudil has been investigated in neurodegenerative and metabolic conditions. A recent clinical trial reported that fasudil is well tolerated and may provide therapeutic benefit in amyotrophic lateral sclerosis [134]. Furthermore, in diabetic patients hospitalized for subarachnoid hemorrhage, fasudil administration was associated with a reduction in proteinuria, suggesting potential renoprotective effects [17].

### 5.2 Isoform-selective inhibition and immunometabolic regulation

Among isoform-selective ROCK inhibitors, belumosudil (KD025) represents the first clinically approved ROCK2-selective inhibitor, originally developed for immunomodulatory therapy. Inhibition of ROCK2 rebalances STAT3/STAT5 signaling and modulates actin-dependent transcriptional programs, thereby limiting Th17 and T follicular helper cell differentiation while enhancing T regulatory and T follicular regulatory subsets. In models of systemic lupus erythematosus and cGVHD, either belumosudil or genetic ROCK2 inhibition alleviated autoimmune inflammation by suppressing IL-17, IL-21, and IFN-γ production, while augmenting IL-10 expression and Treg function [42,43]. Clinical trials confirmed these experimental observations: belumosudil induced durable responses and corticosteroid-sparing effects in refractory cGVHD with a favorable safety profile [135,136], and improved psoriatic pathology through selective suppression of IL-17/IL-23 pathways [137].

Beyond immune regulation, ROCK2 inhibition also contributes to metabolic and mitochondrial homeostasis. Belumosudil administration enhances beige adipocyte formation, thermogenic gene expression, and insulin sensitivity under high-fat diet conditions [138,139]. In diabetic nephropathy, it attenuates ROCK2 activity and NF-κB signaling in the kidney, reducing albuminuria and glomerular sclerosis [40]. ROCK2 blockade further restores FUNDC1-dependent mitophagy in osteoarthritis [126] and diminishes NADPH oxidase-driven ROS generation in monocytes by preventing p47^phox^ phosphorylation [140].

Despite extensive research, no clinically validated ROCK1-selective inhibitors are currently available. Nevertheless, preliminary studies indicate that the natural flavonoid myricetin may exhibit ROCK1-inhibitory activity, potentially ameliorating diabetic glomerulosclerosis by suppressing the ROCK1/ERK/p38 signaling cascade and mitigating mitochondrial oxidative stress [141].

Representative ROCK inhibitors in clinical and experimental development are summarized in Table 2.

**Table 2:** **Clinical and experimental ROCK inhibitors: mechanisms, target organs, and translational status.** cGVHD; chronic graft-versus-host disease, Drp1; dynamin-related protein 1, MLC; myosin light chain, NF-κB; nuclear factor-κB, NET; norepinephrine transporter, ROS; reactive oxygen species, SAH; subarachnoid hemorrhage, STAT; signal transducer and activator of transcription.

Agent	Selectivity	Clinical Status	Mechanism	Target Organ	Key Relevance
**Fasudil**	Pan-ROCK	Approved (SAH vasospasm)	↓MLC phosphorylation; ↓Drp1 phosphorylation; anti-fibrotic	Vasculature; nervous system; kidney	Clinical proof of systemic ROCK inhibition
**Belumosudil**	ROCK2-selective	Approved (cGVHD)	↓STAT3/NF-κB; anti-fibrotic	Immune cells; skin; lung; kidney (preclinical)	Isoform-selective therapeutic strategy
**Ripasudil**	Pan-ROCK	Approved (glaucoma, Japan)	Cytoskeletal modulation	Ocular outflow pathway	Chronic clinical use of ROCK inhibition
**Netarsudil**	Pan-ROCK	Approved (glaucoma)	Cytoskeletal modulation	Ocular outflow pathway	ROCK + NET inhibition
**Y-27632**	Pan-ROCK	Preclinical	↓MLC phosphorylation; ↓Drp1 phosphorylation; ↓ROS; anti-fibrotic	Multiple organs	Widely used experimental ROCK inhibitor

### 5.3 Metabolic drugs indirectly targeting ROCK signaling

Statins, inhibitors of HMG-CoA reductase, not only lower cholesterol but also block the synthesis of isoprenoid intermediates such as geranylgeranyl pyrophosphate. This action prevents RhoA from localizing to the plasma membrane and becoming activated, thereby reducing downstream ROCK signaling. In endothelial cells, statins stabilize eNOS mRNA, enhance Akt-dependent eNOS phosphorylation, and increase nitric-oxide bioavailability, providing vascular protection that is independent of lipid lowering [142]. Yet, statins also affect other small GTPases, including Rac1 and Cdc42, which have distinct roles in maintaining podocyte architecture and slit-diaphragm integrity. Consequently, broad upstream inhibition can produce context-specific outcomes. Indeed, podocyte-specific loss of Cdc42 leads to early proteinuria and glomerulosclerosis, whereas deletion of Rac1 shows variable effects depending on the injury model [143]. Thus, selective targeting of the RhoA/ROCK branch may allow greater therapeutic precision.

Glucagon-like peptide-1 (GLP-1)-based therapies also influence ROCK activity. In diabetes-associated vascular dysfunction, the GLP-1 receptor agonist (GLP-1RA) and the DPP-4 inhibitor suppress the AGE/RAGE-RhoA/ROCK–NF–κB axis while activating AMPK, thereby improving endothelial function and diminishing oxidative stress [67]. In diabetic cardiomyopathy, exendin-4 alleviates lipotoxic injury by inhibiting the ROCK/PPARα pathway and restoring cardiac mitochondrial metabolism [144]. GLP-1RA also enhances insulin secretion in β-cells through PKA-mediated inhibition of RhoA/ROCK and actin depolymerization [145]. In addition, liraglutide ameliorates erectile dysfunction in diabetic rats by downregulating RhoA/ROCK2 signaling and oxidative stress while promoting autophagy [146].

Taken together, these pharmacologic and metabolic interventions underscore the therapeutic versatility of ROCK modulation. Future strategies may integrate isoform-selective inhibition with metabolic or antioxidant therapies to achieve precise restoration of mitochondrial redox homeostasis.

## 6 Future perspectives

The past decade has established ROCK signaling as a pivotal regulator of mitochondrial dynamics, redox homeostasis, and metabolic resilience. Nevertheless, the path toward clinical translation remains incomplete, and several issues merit closer attention. Although pan-ROCK inhibitors such as fasudil have demonstrated therapeutic potential, their lack of isoform specificity narrows the therapeutic window. A major unresolved challenge is the development of selective ROCK1 inhibitors. Because ROCK1 and ROCK2 share >90% amino acid identity in the kinase domain, structure-guided design around the ATP-binding cleft inevitably yields compounds with dual activity. Overcoming this obstacle will likely require alternative strategies, including the identification of allosteric pockets unique to ROCK1, the use of covalent or bitopic inhibitors, or the application of emerging degrader technologies that exploit small differences in surface topology rather than ATP-site affinity.

Another priority is the establishment of reliable biomarkers that allow real-time assessment of ROCK activity in patients. Phosphorylation of MYPT1 in circulating leukocytes has been proposed as a surrogate marker, but its dynamic range, tissue specificity, and responsiveness to selective isoform inhibition remain poorly defined. Prospective studies comparing p-MYPT1 with organ-level mitochondrial parameters, metabolic flux, and clinical endpoints would clarify its value as a pharmacodynamic or stratification biomarker. Identifying isoform-specific readouts—such as mitochondrial fission markers for ROCK1 or immune-related transcriptional signatures for ROCK2—may help refine patient selection in future trials.

From a disease standpoint, the distinction between ROCK1-driven mitochondrial pathology and ROCK2-mediated immunofibrotic processes suggests that isoform-targeted intervention could be matched to specific clinical phenotypes. Experimental models indicate with increasing evidence that ROCK1 is a critical upstream regulator of mitochondrial fragmentation, excessive ROS generation, and metabolic inflexibility in diabetes, obesity, and heart failure. These observations argue for systematic exploration of ROCK1 inhibition in metabolic and cardiorenal disorders, provided that cytoskeletal perturbations can be minimized through isoform-selective or tissue-targeted approaches.

Finally, several approved drugs—including statins, GLP-1 receptor agonists, and certain polyphenols—exhibit partial suppression of RhoA-ROCK signaling. Their capacity to modulate ROCK activity indirectly raises the possibility of combination therapy or drug repurposing to achieve graded, context-dependent pathway inhibition. Taken together, the next phase of ROCK biology will depend on advancing chemical tools that discriminate between ROCK isoforms, validating robust biomarkers of pathway activity, and integrating cell-type-specific ROCK modulation into therapeutic strategies aimed at restoring mitochondrial integrity and metabolic homeostasis.

## Declaration of generative AI in scientific writing

After completing the manuscript, the authors used AI-assisted tools (including ChatGPT) solely for language proofreading and to improve clarity and readability. All scientific content, interpretation, and conclusions were developed and critically reviewed by the authors, who take full responsibility for the accuracy and integrity of the work.

## Funding

This work was supported by 10.13039/501100001691JSPS KAKENHI Grant Number 23K07709, 21K20914, 20K08645, and 18K15985 (to Yosuke Nagai and Keiichiro Matoba).

## CRediT authorship contribution statement

**Yosuke Nagai:** Conceptualization, Visualization, Writing – original draft, Writing – review & editing. **Keiichiro Matoba:** Conceptualization, Supervision, Writing – review & editing. **Rimei Nishimura:** Conceptualization, Supervision, Writing – review & editing.

## Declaration of competing interest

The authors declare the following financial interests/personal relationships which may be considered as potential competing interests: R.N. has received lecture fees from Sanofi, Medtronic Japan, Nippon Boehringer Ingelheim, Teijin Pharma, Kissei Pharmaceutical, Eli Lilly Japan, Novo Nordisk Pharma, Astellas Pharma, Abbott, Sumitomo Pharma, AstraZeneca, Kowa, and Ono Pharmaceutical. R.N. has also received research funding (commissioned and collaborative research) from Mitsubishi Electric, Kowa, and Sanofi.

## Data Availability

No data was used for the research described in the article.
